# Supplementary material for: Ultrastrong magnon-magnon coupling and chiral spin-texture control in a dipolar 3D multilayered artificial spin-vortex ice
Source: Nat Commun. 2024 May 14;15:4077. doi: 10.1038/s41467-024-48080-z (PMC11094080; doi:10.1038/s41467-024-48080-z)
Supplement: Supplementary file 1 — Supplementary Information [file 41467_2024_48080_MOESM1_ESM.pdf]

Supplementary Information - Ultrastrong Magnon-Magnon Coupling and Chiral Spin-Texture Control in a Dipolar 3D Multilayered Artificial Spin-Vortex Ice

Troy Dion[1,*], **Kilian D. Stenning**[2,3], **Alex Vanstone**[2], **Holly H. Holder**[2], **Rawnak Sultana**[4], **Ghanem Alatteili**[5], **Victoria Martinez**[5], **Mojtaba Taghipour Kaffash**[4], **Takashi Kimura**[1], **Rupert F. Oulton**[2], **Will R. Branford**[2,8], **Hidekazu Kurebayashi**[3,6,7], **Ezio Iacocca**[5], **M. Benjamin Jungfleisch**[4], **and Jack C. Gartside**[2,8,*]

[1]Solid State Physics Laboratory, Kyushu University, Japan
[2]Blackett Laboratory, Imperial College London, United Kingdom
[3]London Centre for Nanotechnology, University College London, United Kingdom
[4]Department of Physics and Astronomy, University of Delaware, Newark, DE19716, USA
[5]Center for Magnetic Nanostructures, University of Colorado Colorado Springs, Colorado Springs, CO 80918, USA
[6]Department of Electronic and Electrical Engineering, University College London, London, UK
[7]WPI Advanced Institute for Materials Research, Tohoku University, Sendai, Japan
[8]London Centre for Nanotechnology, Imperial College London, United Kingdom
[*]Corresponding author e-mails: troy.dion@phys.kyushu-u.ac.jp, j.carter-gartside13@imperial.ac.uk

ABSTRACT

Strongly-interacting nanomagnetic arrays are ideal systems for exploring reconfigurable magnonics. They provide huge microstate spaces and integrated solutions for storage and neuromorphic computing alongside GHz functionality. These systems may be broadly assessed by their range of reliably accessible states and the strength of magnon coupling phenomena and nonlinearities.
Increasingly, nanomagnetic systems are expanding into three-dimensional architectures. This has enhanced the range of available magnetic microstates and functional behaviours, but engineering control over 3D states and dynamics remains challenging.
Here, we introduce a 3D magnonic metamaterial composed from multilayered artificial spin ice nanoarrays. Comprising two magnetic layers separated by a non-magnetic spacer, each nanoisland may assume four macrospin or vortex states per magnetic layer. This creates a system with a rich 16^N microstate space and intense static and dynamic dipolar magnetic coupling.
The system exhibits a broad range of emergent phenomena driven by the strong inter-layer dipolar interaction, including ultrastrong magnon-magnon coupling with normalised coupling rates of $\frac{\Delta f}{v} = 0.57$, GHz mode shifts in zero applied field and chirality-control of magnetic vortex microstates with corresponding magnonic spectra.

Supplementary Note 1 - Programmable deactivation of ice rules

Intriguingly, the strong flux-closure in the antiparallel state lifts the energy penalty on traditionally energetically-unfavourable artificial spin system vertex states, so-called 'monopole' excitations[1,2]. While typical square-geometry artificial spin systems often claim 2^N available states, 62.5% of these states are unfavourable monopole states with sufficiently large energy penalty that they are rarely observed. These monopole-like vertices have net magnetic charges of 2 and 4, and are termed 'type 3' and 'type 4' vertices respectively. The remaining lower-energy 'type 1' and 'type 2' vertex states satisfy the 'ice rules'[3] with equal numbers of positive and negative magnetic charge at each vertex, giving zero net magnetic charge. This leads to a substantially reduced ice-rule obeying microstate space of $\frac{3}{\sqrt{2}}^N$. Schematics of the four vertex types with positive and negative magnetic charge illustrated by red and blue circles respectively are shown at the bottom of figure 4i), with the possible number of configurations and percentage of the total microstate space given below.

Here, we have the freedom to reconfigurably deactivate the ice rules on demand via the antiparallel macrospin state. Islands

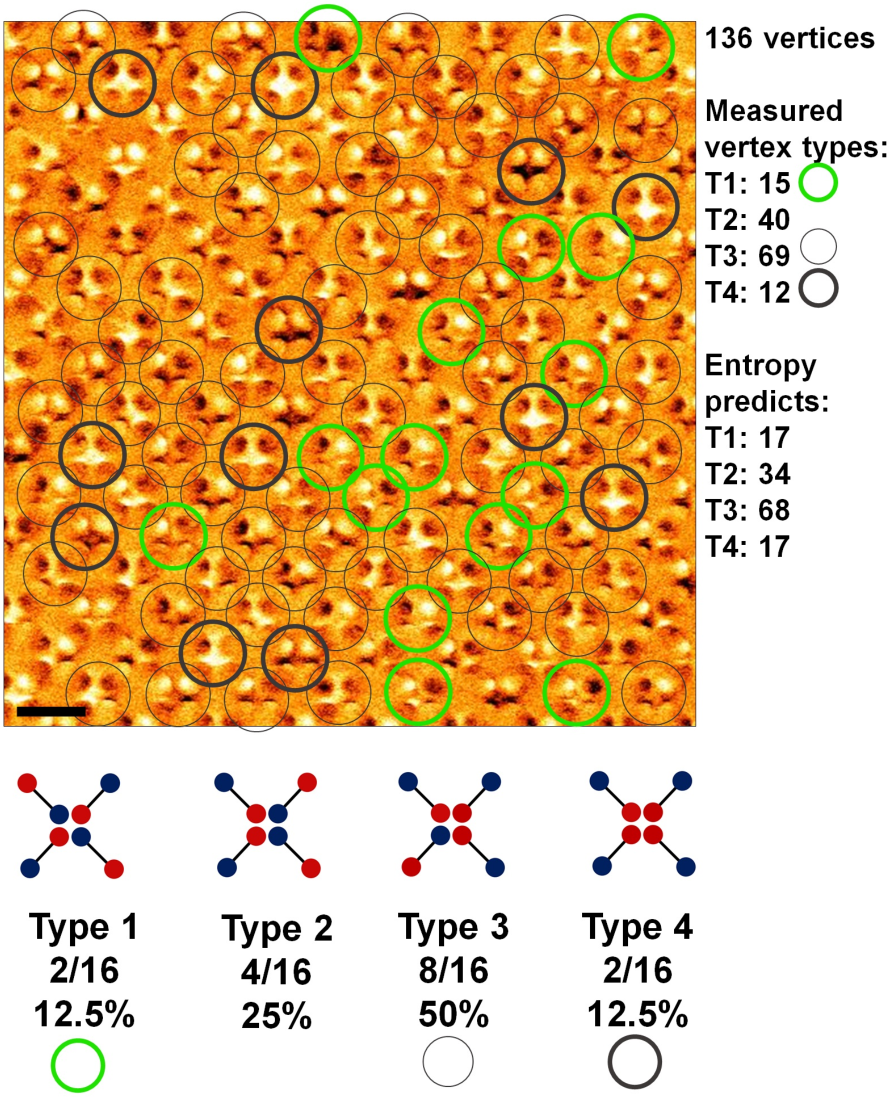

Supplementary Figure 1. Deactivation of ice rules in antiparallel macrospin state. Vertex populations are observed as predicted by entropy, with no forbidding of type 3 (light blue circle) and type 4 (thick blue circle) vertex states. Type 4 vertex states are accompanied by strong opposite-polarity charge emanating into the vertex gap from the lower magnetic layer.

in the antiparallel macrospin state have relatively weak coupling to neighbouring islands due to their inter-layer flux closure. This should lead to a weakening or effective deactivation of the ice rules, allowing access to the full microstate space and vertex populations which closely match the populations predicted by entropy, eg. 12.5% T1, 25% T2, 50% T3 and 12.5% T4[1]. This has high technological relevance, with the restriction on bit-patterned magnetic microstates at increasing array density affecting many proposed applications which leverage the ability to write data into such arrays eg. magnetic storage and neuromorphic computation schemes.

To examine this we prepared a 288 nanoisland array (136 vertices) and analysed MFM of an AC-demagnetised antiparallel macrospin state, shown in figure 4i) with vertex types labelled. The AC-demagnetisation process samples low-energy states of the system and can be considered an effective thermal anneal[4]. From entropic considerations in a system with no forbidden vertex types due to ice-rules, one expects 17 T1, 34 T2, 68 T3 and 17 T4 vertices in an array of this size. We experimentally observe 15 T1, 40 T2, 69 T3 and 12 T4 vertices - remarkably close to entropic predictions with an unsurprising slight bias towards the lower energy T1 and T2 vertices. Such an observation in a strongly-interacting, 125 nm vertex gap artificial spin system is unprecedented, especially as this effective ice-rule deactivation is reconfigurably programmable via microstate selection. This result demonstrates that the 3D magnonic metamaterial architecture presented here truly allows access to the entire microstate space, without the strict state access restrictions placed on conventional artificial spin systems by the ice

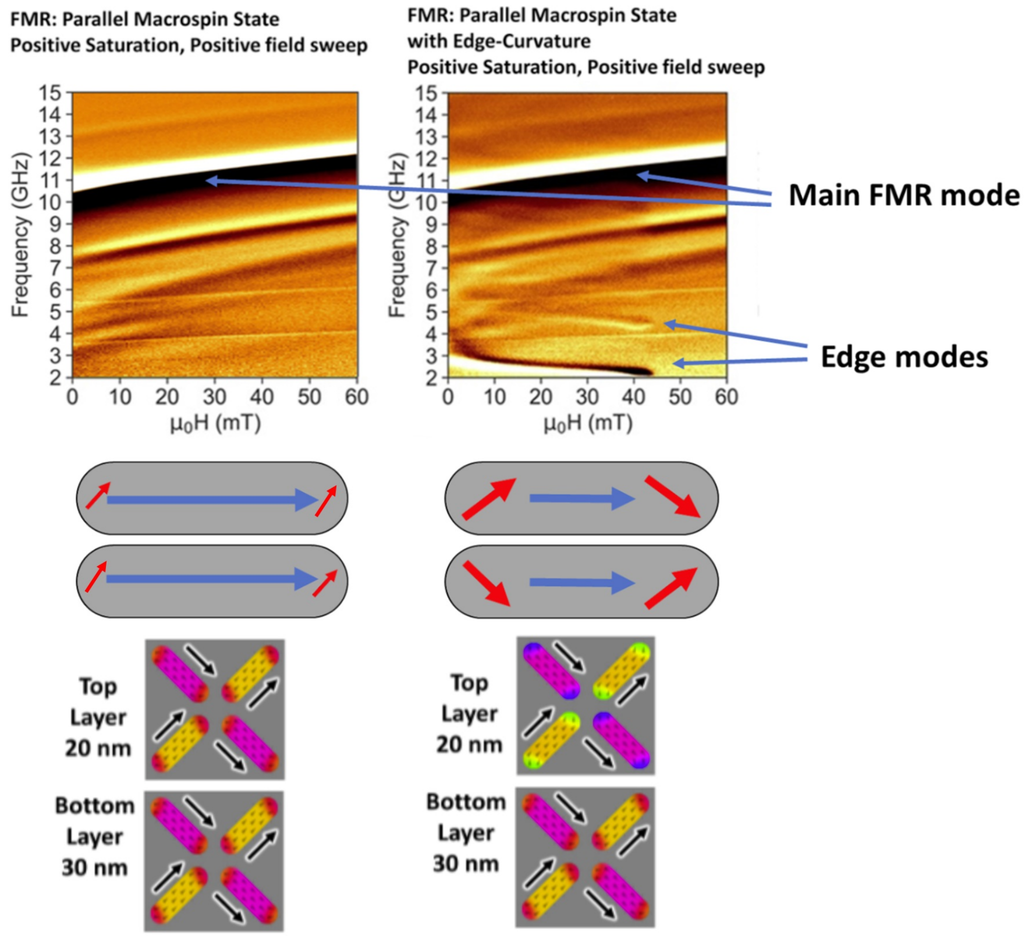

Supplementary Figure 2. Demonstration of reconfigurable edge-mode activation.
a) Experimental FMR of positively-saturated parallel macrospin state, swept 0-60 mT in positive field-sweep direction.
b) Experimental FMR of positively-saturated parallel macrospin state with strong edge-curvature present, swept 0-60 mT in positive field-sweep direction. Edge-curvature state is prepared via negative saturation, +40 mT to prepare chiral edge-curved state by going from negative parallel, positive antiparallel then +P. The edge-curvature state is prepared when switching out of the anti-parallel state and then remaining below the threshold field of 41-44 mT, above which the edge-curvature straightens out.

rules. The ability to access full state spaces at dense array packings has been proposed as a benefit of antiferromagnets and synthetic antiferromagnets. Here, we demonstrate that the same benefits are available within reprogrammable dipolar-coupled 3D nanoarrays. Three-dimensional nanomagnetic systems are often mentioned as means to study frustration by enabling more freedom in spin network design, here we show that 3D systems can also aid studies of frustration by providing a switch to reconfigurably lift the rules of frustration in a system.

Supplementary Note 2 - Reconfigurable edge-mode activation

Here we demonstrate an example of how the edge-curvature state and its strong associated low-frequency 2-3 GHz mode may be reconfigurably activated. Supplementary fig. 2a) shows experimental FMR of a positively-saturated parallel macrospin state, swept 0-60 mT. A dominant mode is observed around 10-11 GHz. The 2-3 GHz region is relatively empty save for a weak sub-mode of the dominant mode (around 3.5 GHz at 0 field, positive field-frequency gradient). We now prepare the edge curvature state to activate the low frequency mode - this is done by negatively saturating, applying +40 mT to transition through the anti-parallel state to a positive parallel macrospin state. As the system switches out of the antiparallel state, the edges strongly curve and lock in an exaggerated S/C-state to minimise the unfavourable stray field condition of aligning like-charge poles above each other in the two layers.

At $\sim$44 mT the edge-curvature mode straightens out and the system assumes a pure parallel macrospin state. The FMR sweep in fig. 4a) mirrors the same forward field sweep on positively-magnetised parallel macrospin mode in fig. 3e), with

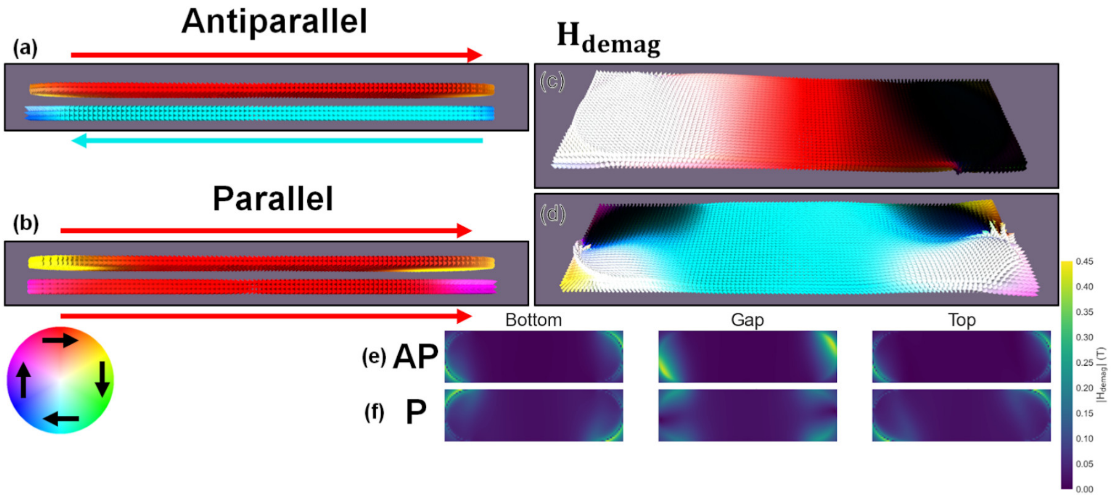

Supplementary Figure 3. The demagnetisation field between bilayer single islands.
a) and **b)** show the MuMax3 magnetisation in the antiparallel (AP) and (P) configuration respectively.
The colourwheel maps demagnetisation field direction in 3D space with the shading (black to white) showing the $\hat{z}$-components and hue showing the $\hat{x}\hat{y}$-components. The magnitudes are shown in (e) and (f).
c) shows the demagnetisation field in the spacer layer region for the antiparallel (AP) macrospin configuration. The opposite poles on the two ends generate a strong z demagnetisation field that extends beyond the spacer layer, looping up from the bottom layer to the top layer (left hand side) and down from the top layer into the bottom layer (right hand side). There is a slight demagnetisation field in the positive x direction (towards the right of the image) due to the unequal thickness of the top and bottom bar.
d) shows the demagnetisation field in the parallel (P) macrospin configuration. Here, like-charged poles at each end of the nanoisland create equal but opposite demagnetisation field within the spacer layer, and equal but opposite demagnetisation field in $\hat{z}$ in the space between magnetic layers, resulting in a net demagnetisation field in the xy-plane while very little in z beyond the bar.
The magnitude of the demagnetisation field in both the antiparallel (AP) and parallel (P) in the bottom, gap and top layers is shown in (e, f) respectively.

the noticeable difference of the striking edge-curvature mode at 3 GHz and its higher harmonic at 5 GHz. The ability to programmably activate and deactivate these low-frequency magnon channels while leaving the higher-frequency macrospin mode unaffected demonstrates the highly reconfigurable magnon mode structure offered by this 3D architecture.

We now repeat the 0-60 mT and a very strong mode is seen 2-3 GHz with opposite differential contrast than the dominant mode (light-to-dark vs dark-to-light).

Such edge-curvature modes are typically very weak and challenging to resolve in such nanopatterned arrays, with most magnon/spin-wave measurements failing to resolve them[5–8] and most studies concerning them using micromagnetic simulation or theory[9–12]. The strong edge-mode amplitude here is due to the higher degree of curvature induced by 3D inter-layer coupling, as discussed above.

Supplementary Note 3 - Demagnetisation field profiles in single multilayered islands and array vertices

In 3D multilayer nanoislands the demagnetisation field between the individual layers depends strongly on whether the islands are in a parallel or antiparallel macrospin state. Supplentary figures 3, 4 and 5 show cartoon schematic and MuMax3 visualisations of the relative demagnetisation field profiles for the parallel and antiparallel macrospin states, discussion provided in figure captions.

Supplementary Note 4 - Optical and acoustic relative mode frequencies

Optical modes are often defined to be higher-frequency than acoustic modes when oscillators are coupled only at one end, as in the 1D-chain of atoms often used to describe acoustic and optical phonons or indeed single-layer artificial spin systems where adjacent islands couple in-plane across vertices[5,7]. Here, both ends of the oscillators are equally coupled due to the 3D multilayered architecture, which gives an out-of-phase optical mode at lower frequency than the in-phase acoustic.

A good analogy may be found in the hybridisation behaviour of surface plasmon resonances in metallic nanoparticles. A

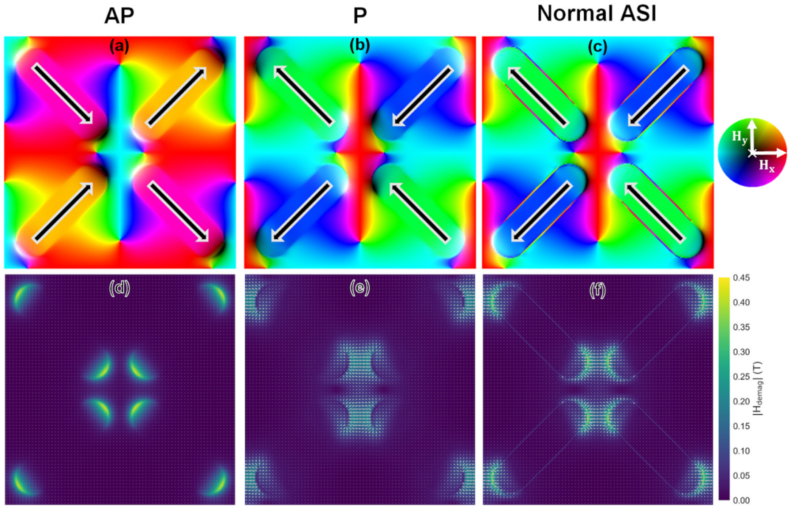

Supplementary Figure 4. The demagnetisation field between bilayer ASI. First row shows the demagnetisation field direction as a heatmap with the shade (black into the plane) showing the $\hat{z}$-component and hue showing the $\hat{x}\hat{y}$-component. The second row shows the magnitude of the demagnetisation field and the quiver plot shows the demagnetisation field in the $\hat{x}\hat{y}$-directions. **a)** shows the MuMax3 simulated demagnetisation field integrated in the spacer between the top and bottom layer (30 – 60 nm) while in the AP state. There is strong coupling between the opposite poles in the bilayer generating a strong z-demagnetisation field and very little lateral coupling as shown by **d)**. This breaks the symmetry leading to strong coupling between the two left bilayer islands with the same downward demagnetisation field and vice versa for the right side. **b)** shows the demagnetisation field in the P state where the like charges make the demagnetisation field much more similar to what is seen in single layer ASI **c)**. The nanobars are laterally coupled shown in **e)** and **f)** The arrows show the net moment of the bars (ie the magnetisation of the bottom layer).

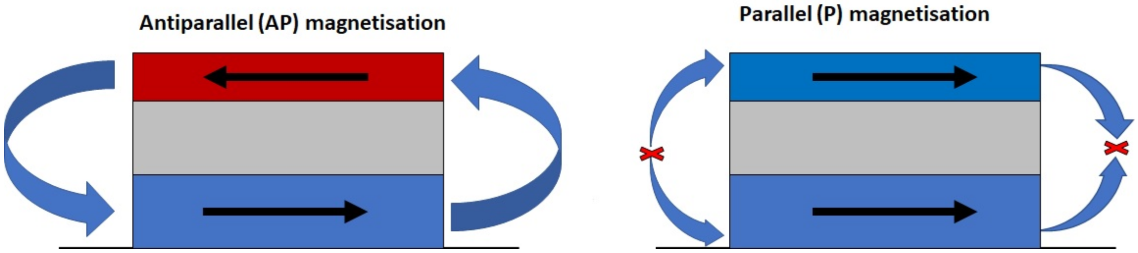

Supplementary Figure 5. Schematic of stray field in parallel and antiparallel macrospin states. In the antiparallel (AP) case, dipolar field emanating from one layer sums with the magnetisation in the other layer, increasing the internal field and hence raising the magnon mode frequency. However, outside of the nanoisland the stray fields of each layer cancel each other - hence in the AP nanoisland case the internal dipolar field is strong, and the external dipolar field is weak.
In the parallel (P) case, the dipolar field emanating from one layer opposes the magnetisation in the other layer. However, outside of the nanoisland the stray fields of each layer sum - in the P case the external dipolar field is strong.

detailed investigation of various geometries between neighbouring nanoparticles/nanoislands and the effect of these geometries on the relative frequencies of the acoustic and optical modes is given by Davis et al[13].

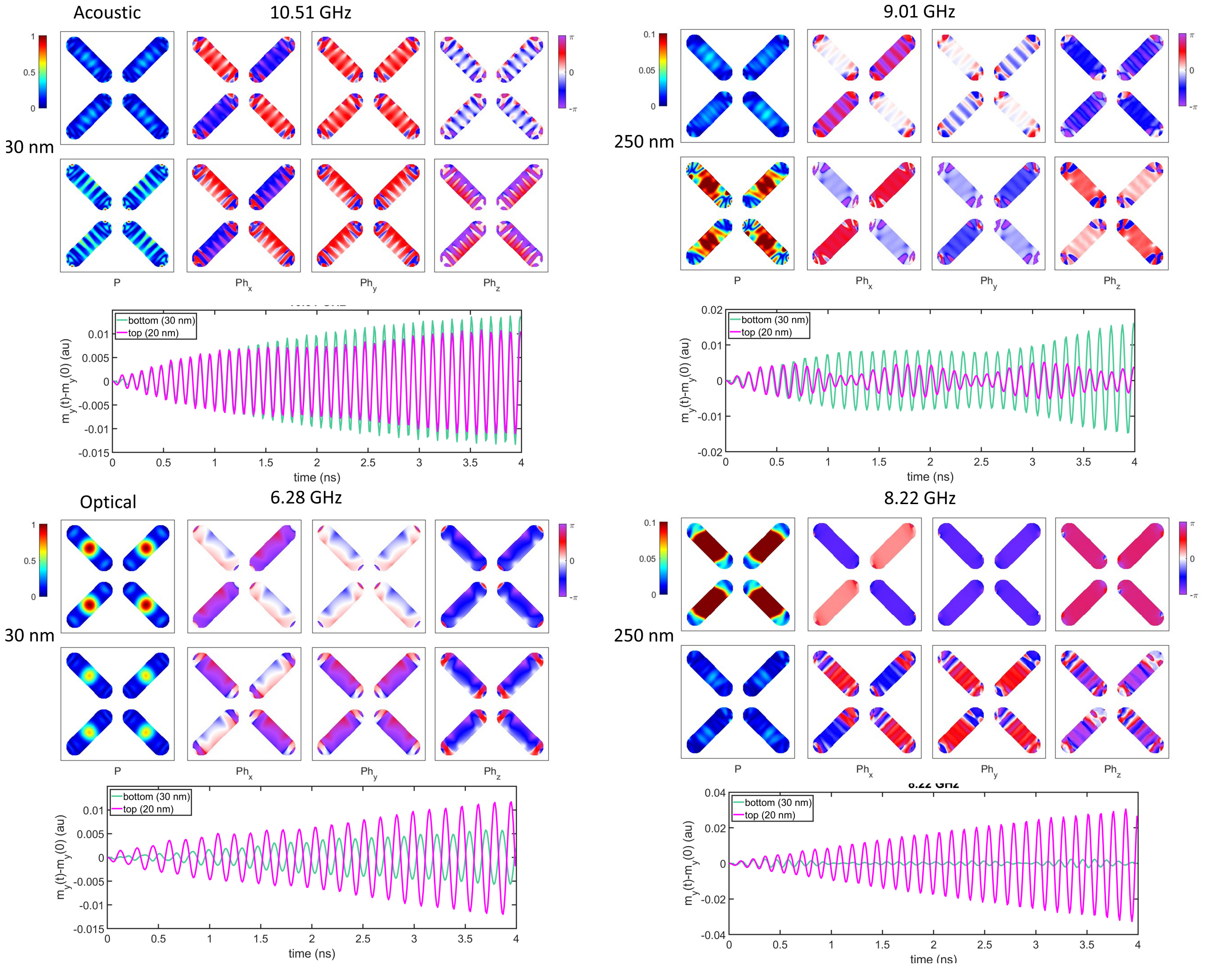

Supplementary Figure 6. Spatial power and phase mode profiles for the 30 nm non-magnetic spacer (left column) and 250 nm non-magnetic spacer (right column). In the 30 nm case, acoustic (10.51 GHz) and optical (6.28 GHz) modes are seen with in-phase/out-of-phase inter-layer oscillations respectively. In the 250 nm case, there is negligible magnon coupling and modes are not hybridised. Panels below $\hat{y}$-component of magnetisation of an island in the top and bottom layers when excited by a monochromatic sine-wave RF field excitation.

Supplementary Note 5 - RF field geometry and magnon mode coupling

The choice of RF field geometry relative to the sample magnetisation and applied field direction has a strong effect on which magnon modes can couple to the RF field and be excited. Supplementary figure 8 shows MuMax3 simulations of a range of RF field directions. For all cases, $\mathbf{H}_{RF}$ is applied along $\hat{x}$ and the nanoarray is in an antiparallel state, thick layer magnetised in positive $\hat{x}$ and thin layer magnetised in negative $\hat{x}$. The experimental FMR spectra (bottom row, centre) has a $\hat{y}$ RF field.

Acoustic and optical modes are observed for all RF field geometries except for $\hat{y}$, with no optical mode observed in the experimental FMR.

Interestingly, in multilayered thin-film geometries such as synthetic antiferromagnets - a z RF field does not satisfy the correct geometric conditions to couple to the optical mode. The fact that we observe it here in simulation and experiment (the BLS experiment is excited by a z RF field) shows that by nanopatterning into our 3D metamaterial architecture, we have expanded the range of RF field mode coupling geometries beyond what is possible in conventional thin-film systems.

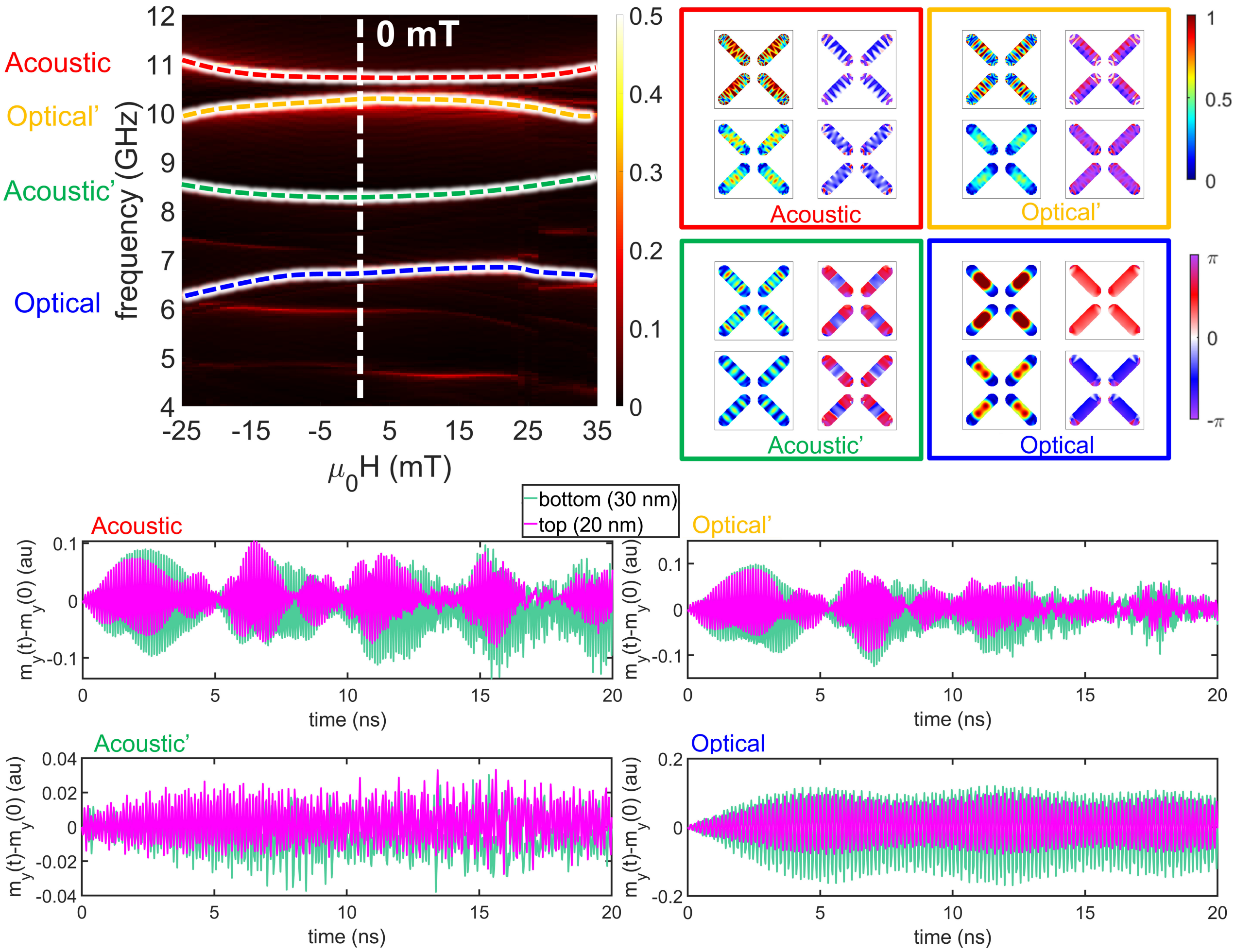

Supplementary Figure 7. Examination of higher-order modes which appear for thinner non-magnetic spacer layers (40-20 nm). Simulated field-frequency heatmap (40 nm spacer case) shows four modes, primary acoustic and optical modes (red and blue), and higher-order modes related to them, labelled acoustic’ and optical’ (green and yellow). Power and phase spatial mode profiles are shown for all four modes, taken at 0 mT. The magnetisation vs. time trace of the acoustic and optical’ modes show a beating with a period consistent with the frequency gap size between the acoustic and optical’ modes, additionally the beating pattern amplitude envelope of the two modes has similar shape. The higher order modes are resolved experimentally, seen in fig. 3g).

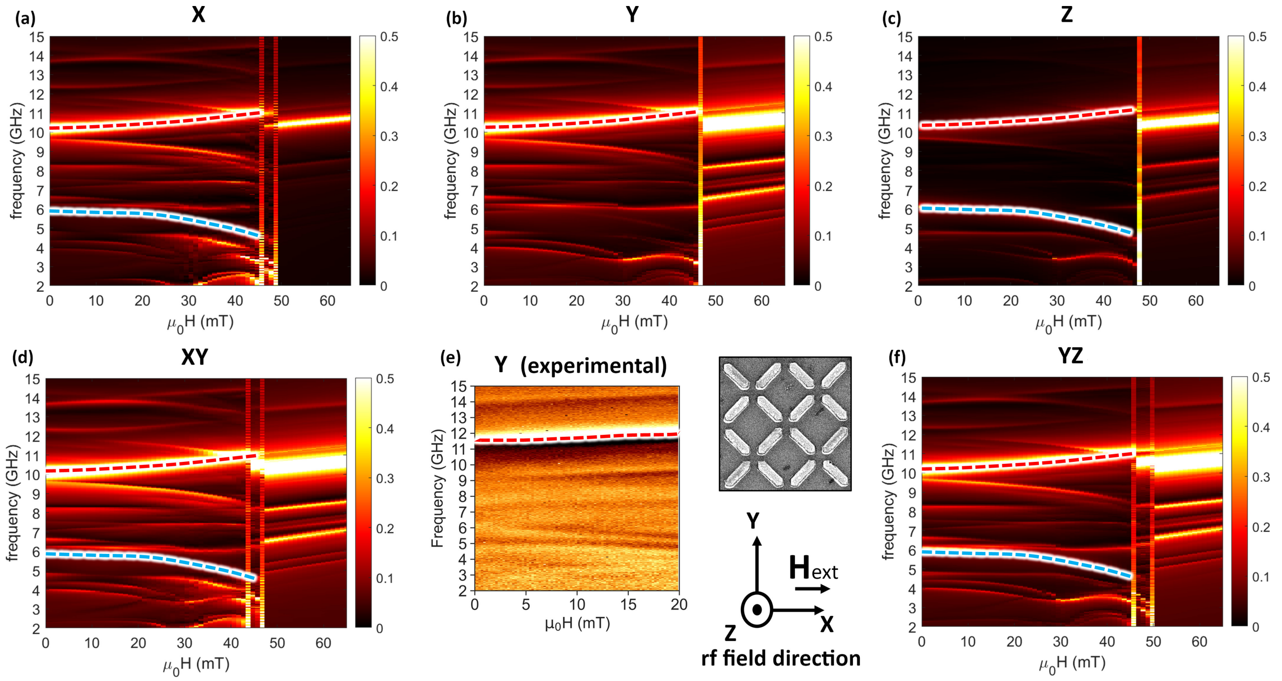

Supplementary Figure 8. MuMax3 simulated FMR field sweeps for different RF field directions. Field is applied in the x-direction for all spectra, with RF field applied in **a)** X, **b)** Y, **c)** Z, **d)** XY and **f)** YZ directions. (b) most closely resembles the experimental result shown in **e)**. **(e)** Same as fig. 3 (g) but with perpendicular DC external field and RF field (illustrated by the inset) demonstrating optical mode is not excited in this geometry.
The system is initialised in the AP state at 0 field and sweept up to 65 mT for each RF field configuration. System switches into parallel state around 48 mT. Typical FMR with perpendicular RF field does not efficiently excite the optical mode. Some x or z component of the RF rf field is required to excite. That the optical mode is observed with a purely z RF field is notable - for continuous multilayer films such as SAFs a Z RF field is unable to couple to the optical mode. The nanopatterning and different thickness magnetic layers employed here result in a broken symmetry, allowing a purely Z RF field to couple to and excite the optical mode. Experimental FMR spectra is shown with Y-oriented RF.

Supplementary Note 6 - Simulated analysis of stray dipolar field profiles and magnitudes

We have used MuMax3 for micromagnetic simulations to investigate the profile and magnitude of the stray dipolar field emanating from each magnetic layer in supplementary figure 9. We have simulated two cases: a 30 nm thick NiFe nanoisland (left four columns) and a 20 nm thick NiFe nanoisland (right four columns). We then plot the $\hat{x}$,$\hat{y}$,$\hat{z}$ and quadrature combined $\hat{x}$,$\hat{y}$,$\hat{z}$ components of the stray dipolar field (columns are labelled at the bottom of each column), with each row showing the dipolar field for a 10 nm $\hat{z}$ slice. The substrate is at the bottom of the column, in the 30 nm island case (left four columns) the upper magnetic layer will be situated in the top two rows, in the 20 nm island case (right four columns) the upper magnetic layer will be situated in the top three rows. The upper magnetic layer positions are shown by curly brackets. To clarify, we are only simulating a single magnetic layer at a time to extract the dipolar field from each layer separately.

From the total field column, we see there is a peak field at the position of the adjacent magnetic layer of 60-80 mT in the 30 nm island case, and a peak field of 35-45 mT in the 20 nm island case. These fields are highly spatially nonuniform, concentrated above the nanoisland ends as expected for a dipolar field projected from the poles of a macrospin nanoisland. As the mode is located at the centre of the bar the field here will contribute the most to the shift in frequency, which is lower than at the poles. The field component along the long axis of the nanoislands around the nanoisland centre is 15-17 mT in the case of the 20 nm island and 18-21mT in the case of the 30 nm island, with this variation due to the spatially-nonuniform nature of the dipolar field.

From Fig 3a) we can use the experimental FMR data to look at the amount of spatially-uniform externally-applied magnetic field required to generate a shift of 1 GHz (the difference in mode resonance between the parallel and antiparallel macrospin states).

From a resonance of 11 GHz at 20 mT and a resonance of 12 GHz at 53 mT, we can model the mode frequency/field gradient in this region as 1 GHz / 33 mT using a linear first-order approximation.

The experimentally-measured blueshift in mode-frequency between the parallel and antiparallel states is 1 GHz. The shift

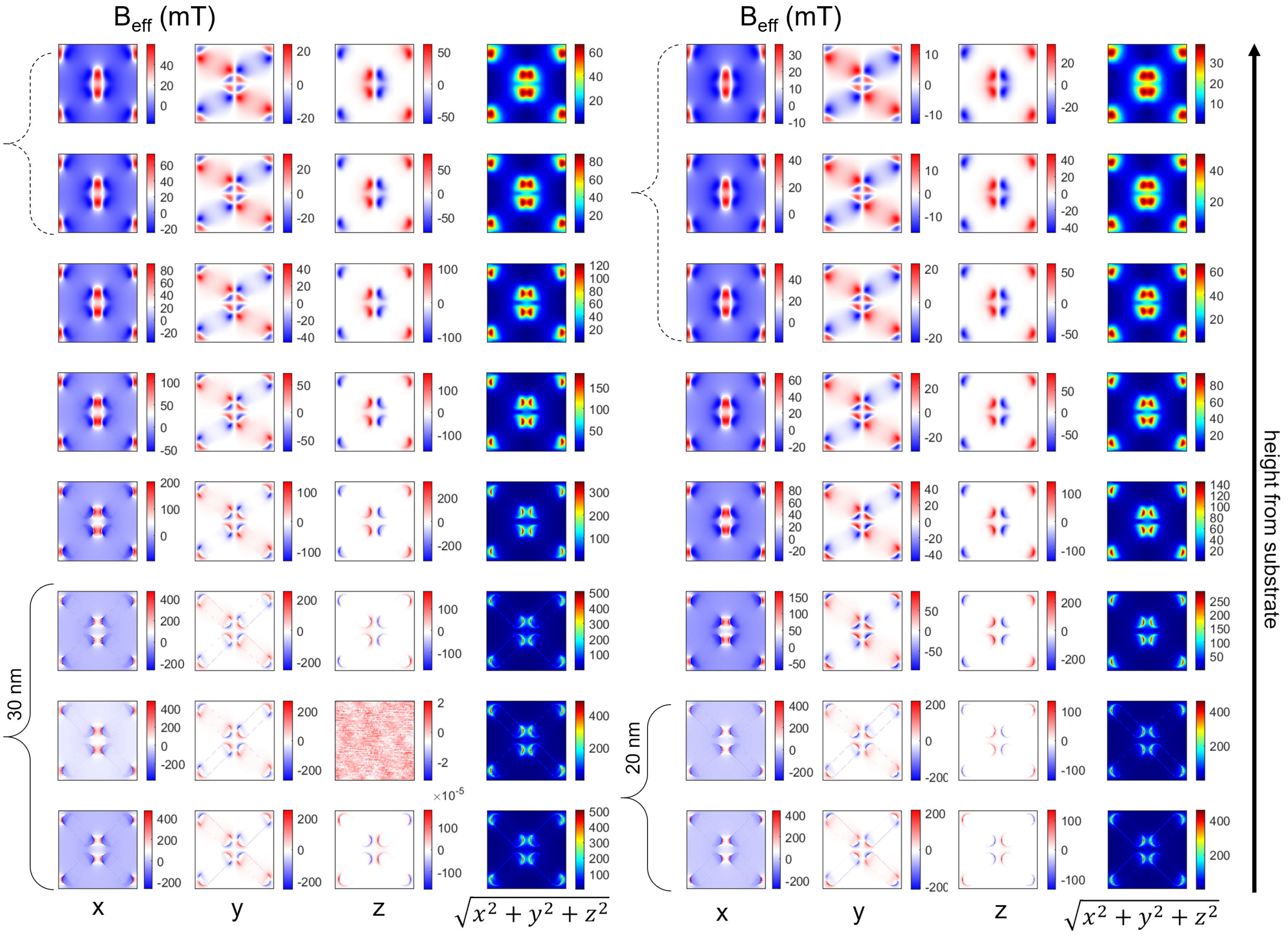

Supplementary Figure 9. Micromagnetic MuMax3 simulation of stray dipolar field profiles projected by a 30 nm NiFe nanoisland (left four columns) and a 20 nm NiFe nanoisland (right four columns). Each column is a different spatial component of the dipolar field, with the total given by quadrature combinations of$\hat{x}$, $\hat{y}$, $\hat{z}$ as shown in the column labels. Each row is a 10 nm z-slice, the substrate is below the bottom row. The position of the magnetic nanoislands are indicated by curly brackets with the 30 nm (left columns) and 20 nm (right columns), the position that the adjacent magnetic layer would occupy is highlighted by the unlabelled top curly brackets for both columns.

in dipolar field between these states is 15-17 + 18-21 mT = 33-38 mT. This is a good match for the experimentally estimated value of 33 mT for a 1 GHz frequency shift.

Supplementary Note 7 - Chiral selectivity

Supplementary figure 10 shows the mechanism for chiral selectivity. One of the magnetic layers is fixed in a macrospin state (shown first in '1').Then, a second $\hat{z}$-separated layer is introduced, with a lateral offset in either $\hat{x}$ (left column) or $\hat{y}$ (right column). The two different layer offset directions result in different overlapping areas between the layers, shown by the shaded regions. These overlapping regions have the strongest inter-layer coupling, and hence determine the overall character of the inter-layer coupling. To lower the overall system dipolar energy, the shaded regions should have opposite magnetisation directions.

In an antiparallel macrospin state this is easy to compute as macrospin magnetisations are collinear throughout each nanoisland. However, when one layer is a vortex the picture becomes slightly more complex - here we consider the net magnetisation of the vortex texture within the shaded overlapping regions. Depending on whether the inter-layer offset is in x or y, the vortex state is forced into either 50/50 clockwise/anticlockwise chiralities ($\hat{x}$ offset for an $\hat{x}$-magnetised macrospin layer) or all clockwise vortices ($\hat{y}$ offset for an $\hat{x}$-magnetised macrospin layer) in order to minimise the overall system energy.

Supplementay figure 12 shows energy difference calculations between all states for different static field directions to determine if core polarity can also be selected. We found no significant energy differences for selecting between polarities for

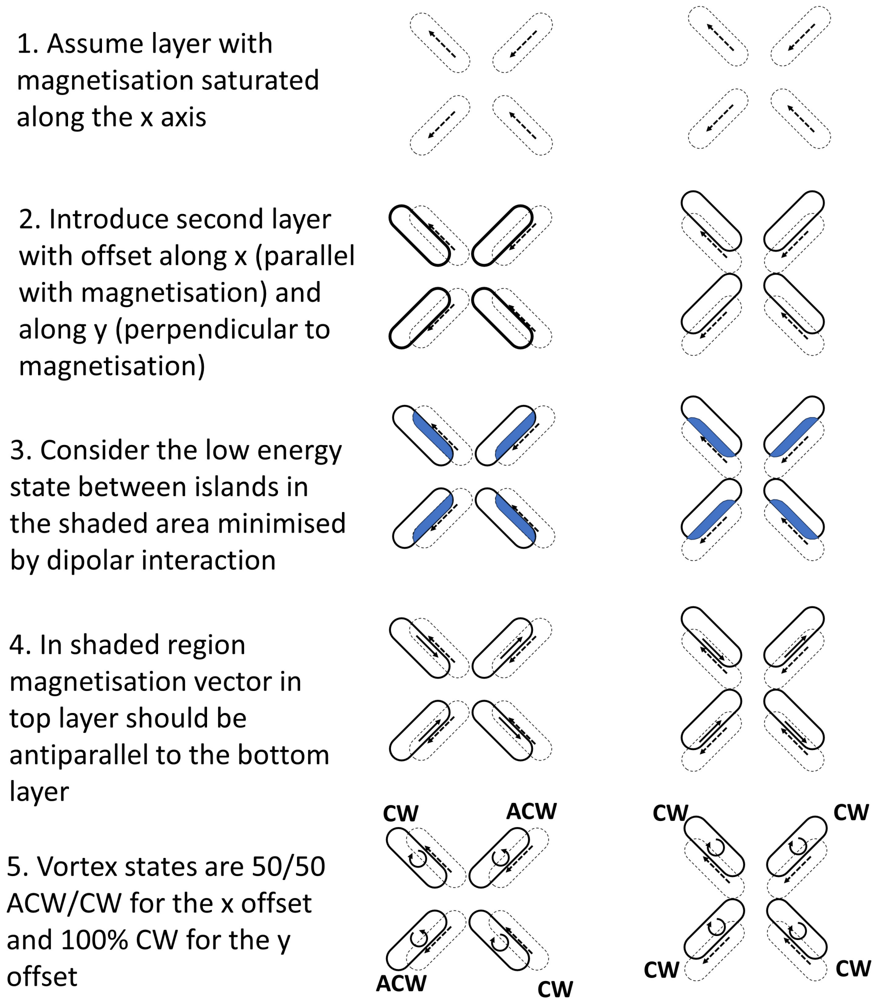

Supplementary Figure 10. Schematic for vortex chiral selectivity mechanism for $\hat{x}$ (left column) and $\hat{y}$ (right column) lateral inter-layer offsets. In both cases one layer is a -$\hat{x}$ magnetised macrospin layer, and the other layer assumes the vortex chiralities required to minimise overall system dipolar/exchange energy.

both opposite circularity and same circularity. The small differences exist (all less then %) and would be impractical to achieve experimentally and other factors such as quenched disorder would add further complications.

References

1. Wang, . R. *et al.* Artificial 'spin ice'in a geometrically frustrated lattice of nanoscale ferromagnetic islands. *Nature* **439**, 303–306 (2006).
2. Ladak, S., Read, D., Perkins, G., Cohen, L. & Branford, W. Direct observation of magnetic monopole defects in an artificial spin-ice system. *Nat. Phys.* **6**, 359–363 (2010).
3. Pauling, L. The structure and entropy of ice and of other crystals with some randomness of atomic arrangement. *J. Am. Chem. Soc.* **57**, 2680–2684 (1935).
4. Nisoli, C. *et al.* Ground state lost but degeneracy found: The effective thermodynamics of artificial spin ice. *Phys. review letters* **98**, 217203 (2007).
5. Gartside, J. C. *et al.* Reconfigurable magnonic mode-hybridisation and spectral control in a bicomponent artificial spin ice. *Nat. Commun.* **12**, 1–9 (2021).
6. Dion, T. *et al.* Tunable magnetization dynamics in artificial spin ice via shape anisotropy modification. *Phys. Rev. B* **100**, 054433 (2019).

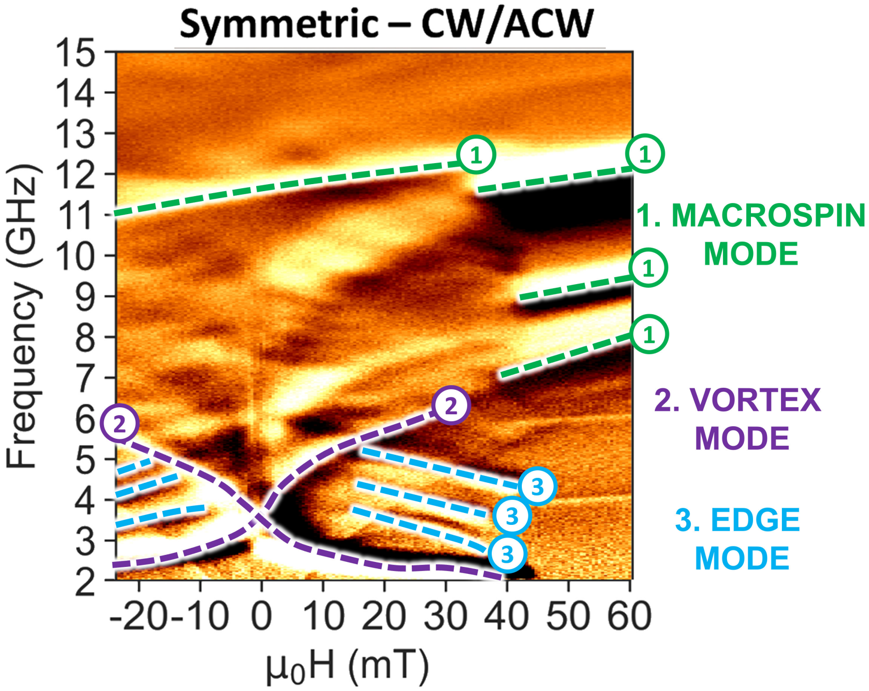

Supplementary Figure 11. Mode assignment for fig. 4i) labelling which magnetic textures each mode originates from

7. Dion, T. *et al.* Observation and control of collective spin-wave mode hybridization in chevron arrays and in square, staircase, and brickwork artificial spin ices. *Phys. Rev. Res.* **4**, 013107, DOI: 10.1103/PhysRevResearch.4.013107 (2022).
8. Kaffash, M. T., Lendinez, S. & Jungfleisch, M. B. Nanomagnonics with artificial spin ice. *Phys. Lett. A* **402**, 127364 (2021).
9. Iacocca, E., Gliga, S. & Heinonen, O. G. Tailoring spin-wave channels in a reconfigurable artificial spin ice. *Phys. Rev. Appl.* **13**, 044047 (2020).
10. Iacocca, E. & Heinonen, O. Topologically nontrivial magnon bands in artificial square spin ices with dzyaloshinskii-moriya interaction. *Phys. Rev. Appl.* **8**, 034015 (2017).
11. Iacocca, E., Gliga, S., Stamps, R. L. & Heinonen, O. Reconfigurable wave band structure of an artificial square ice. *Phys. Rev. B* **93**, 134420 (2016).
12. Slöetjes, S. D., Hjörvarsson, B. & Kapaklis, V. Texture fluctuations and emergent dynamics in coupled nanomagnets. *Phys. Rev. B* **106**, 104405 (2022).
13. Davis, T., Gómez, D. & Vernon, K. Simple model for the hybridization of surface plasmon resonances in metallic nanoparticles. *Nano letters* **10**, 2618–2625 (2010).

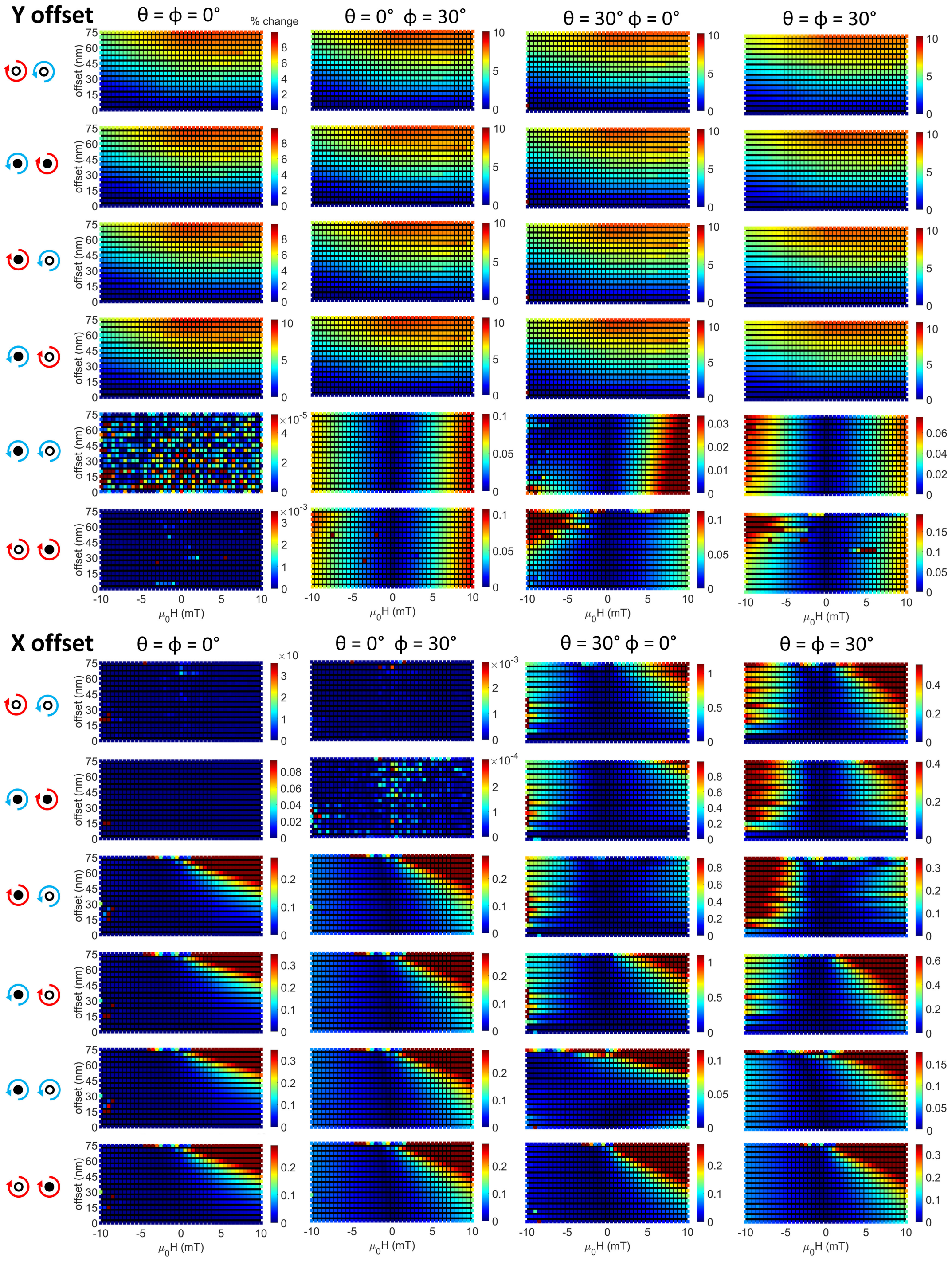

Supplementary Figure 12. Energy difference calculations for all possible circulation and polarity states as a function of layer offset, field magntitude and direction. ϕ and θ are the out-of-plane and in-plane angles respectively.
